# Comparative analysis of two mathematical algorithms for the calculation of computed tomography perfusion parameters in the healthy and diseased pancreas

**DOI:** 10.1002/acm2.13488

**Published:** 2021-12-13

**Authors:** Kehua Pan, Hongqing Wang, Xiaoyu Chen, Xiaocui Ye, Zhao Zhang, Xiao Chen, Xiufen Jia

**Affiliations:** ^1^ Department of Radiology The First Affiliated Hospital of Wenzhou Medical University Wenzhou China; ^2^ Department of Ultrasonics The First Affiliated Hospital of Wenzhou Medical University Wenzhou China

**Keywords:** algorithm, pancreatic perfusion, tomography, X‐ray computed

## Abstract

**Background:**

The maximum slope (MS) and deconvolution (DC) algorithms are commonly used to post‐process computed tomography perfusion (CTP) data. This study aims to analyze the differences between MS and DC algorithms for the calculation of pancreatic CTP parameters.

**Methods:**

The pancreatic CTP data of 57 patients were analyzed using MS and DC algorithms. Two blinded radiologists calculated pancreatic blood volume (BV) and blood flow (BF). Interobserver correlation coefficients were used to evaluate the consistency between two radiologists. Paired *t*‐tests, Pearson linear correlation analysis, and Bland–Altman analysis were performed to evaluate the correlation and consistency of the CTP parameters between the two algorithms.

**Results:**

Among the 30 subjects with normal pancreas, the BV values in the three pancreatic regions were higher in the case of the MS algorithm than in the case of the DC algorithm (*t* = 39.35, *p* < 0.001), and the BF values in the three pancreatic regions were slightly higher for the MS algorithm than for the DC algorithm (*t* = 2.19, *p* = 0.031). Similarly, among the 27 patients with acute pancreatitis, the BV values obtained using the MS methods were higher than those obtained using the DC methods (*t* = 54.14, *p* < 0.001). Furthermore, the BF values were higher with the MS methods than the DC methods (*t* = 8.45, *p* < 0.001). Besides, Pearson linear correlation and Bland–Altman analysis showed that the BF and BV values showed a good correlation and a bad consistency between the two algorithms.

**Conclusions:**

The BF and BV values measured using MS and DC algorithms had a good correlation but were not consistent.

## 1 BACKGROUND

With the continuous expansion of the clinical applications of multi‐slice spiral computed tomography (CT), CT perfusion (CTP) imaging has become increasingly important in the diagnosis, therapeutic monitoring, and prognostic evaluation of pancreatic diseases, especially pancreatic cancer.^1^, ^2^, ^3^, ^4^, ^5^ Two mathematical algorithms are commonly used for the postprocessing of CTP imaging data: the maximum slope (MS) algorithm and the deconvolution (DC) algorithm. Currently, many types of CTP software programs are used in clinical practice, but only two mathematical algorithms are commonly used to process CTP data: the MS and the DC algorithm. The MS algorithm is first proposed by Peters in 1987 and hypothesizes that after the injection of a contrast agent, the MS of the time–density curve (TDC) is reached within a short time without any contrast agent outflow. Thus, blood flow (BF) within the tissues is BF volume = maximum slope of tissue TDC/peak enhancement of the input artery. Blood volume (BV) cannot be directly calculated using the MS method, so an alternative equivalent BV was calculated using the Patlak plot methods.^6^ Miles et al.^7^ successfully applied the method to pancreatic CTP. Cenic et al.^6^ proposed the DC algorithm in 1999. This algorithm converts the time–history data of each pixel position to the corresponding impulse residue function to reflect the amount of contrast agent in the tissues after an intravenous bolus injection of the contrast agent. The BF and BV in the pancreas can be calculated using the DC methods. The MS methods require a high injection rate of the contrast agent (generally 6–8 ml/s); the perfusion values will be underestimated at lower rates. Thus, the clinical applicability of the MS method is limited in patients with cardiac or venous insufficiency. In this study, a total of 11 patients were excluded because of cardiac or venous insufficiency. In contrast, the DC method provided high spatial and temporal resolution, had no special requirement of contrast injection rate, and was less affected by the cardiac function. Furthermore, not many hypothetical assumptions are made in establishing the tissue perfusion model. Thus, the perfusion parameters derived using this method are closer to the actual physiological variables.^8^, ^9^ There are studies on the interrater agreement in CTP studies^10^, ^11^ and the correlation and consistency of the pancreatic CTP values obtained using these two algorithms have become a critical issue. The knowledge of the comparability of different mathematical algorithms is necessary for the standardization of CTP post‐processing methods as well as for extending the clinical applications of pancreatic CTP imaging.^12^ Multiple studies have shown that CTP parameters calculated using MS and DC algorithms differ significantly between patients with head and neck tumors^13^, ^14^, ^15^ and esophageal tumors.^16^ Meanwhile, the clinical applications of pancreatic CTP are applied in acute ischemic stroke,^17^ abdominal organs and diseases,^18^ and various oncologic and nononcological clinical applications.^19^ However, to our knowledge, there are currently no clinical studies investigating MS and DC algorithms used to calculate pancreatic CTP parameters.

Thus, we conducted the present study to comparatively assess the pancreatic CTP data obtained using the MS and DC algorithms in both subjects with normal pancreas and patients with acute interstitial pancreatitis to determine the effect of the mathematical algorithm used on the calculation of pancreatic perfusion parameters.

## 2 METHODS

### 2.1 Patient selection

We retrospectively reviewed the data of two categories of patients: (a) patients for whom CTP imaging of the liver was clinically indicated for the evaluation of hepatocellular carcinoma and who had a normal pancreas; (b) patients for whom CTP imaging of the pancreas was clinically indicated for the grading of acute pancreatitis. All subjects had undergone 320‐slice volume CT between January and August 2015. The exclusion criteria were heart, liver, or renal insufficiency; and respiratory, movement, or other artifacts. This study protocol was approved by the Ethics Committee of the First affiliated hospital of Wenzhou Medical University. The written informed consent was obtained from all patients.

### 2.2 CTP technique

**FIGURE 1:**
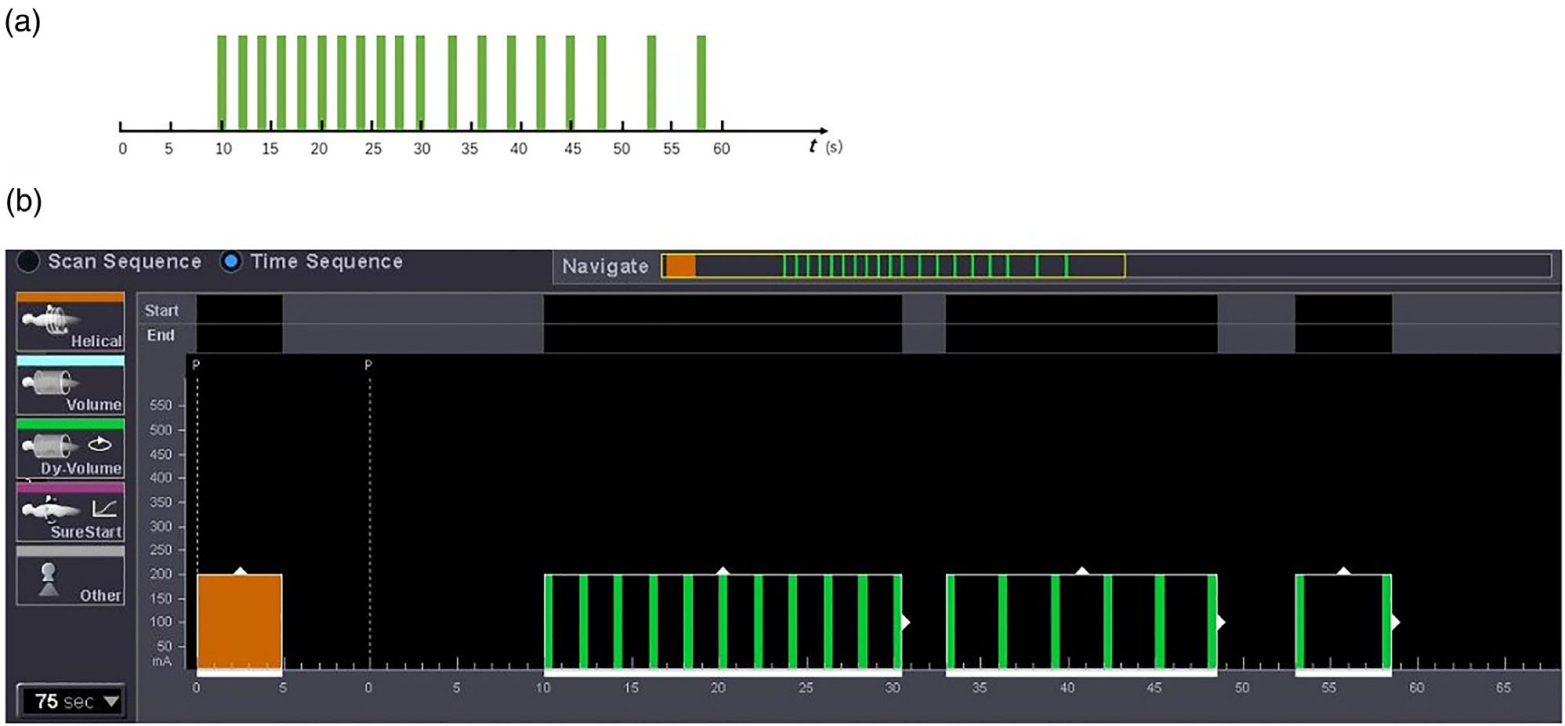
(a and b) The detailed scanning protocol of pancreatic computed tomography perfusion (CTP)

All patients were required to be in a supine position and wear an abdominal compression band for CTP imaging. They were trained to breathe shallowly and regularly to minimize respiratory excursions of the abdominal wall. Pancreatic CTP imaging was performed using a 320‐slice dynamic volume CT scanner (Aquilion One, Toshiba, Tokyo, Japan). A plain scan (120 kVp; 100 mAs; slice thickness, 5 mm; rotation time, 0.5 s) was performed to locate the pancreas. We intravenously injected a nonionic contrast agent (Omnipaque, 350 mg iodine/ml, 1 ml/kg; GE Healthcare, Shanghai, China) at a flow rate of 7 ml/s using a Medrad double‐tube pressure syringe through a 21‐gauge catheter, following by a 30‐ml saline flush. The dynamic perfusion scan began 10 s after the contrast medium injection and the detailed protocols are shown in Figure 1. Next, 19‐phasic volume data were acquired using the AIDR 3D standard reconstruction algorithm. The images were reconstructed with a slice thickness of 0.5 mm and an interval of 0.5 mm, which resulted in 320 images per phasic series.

### 2.3 Image analysis

The MS method assumed that there was no contrast agent outflow in a short time before the TDC reached the MS after injection of the contrast agent. BF per unit volume of tissues was derived as the ratio of the MS of TDC in tissue to the peak of TDC enhancement in the input artery. The DC method converted the time–history data of each pixel position into the corresponding residual function, which reflected the amount of contrast agent in the tissue over time after intravenous mass injection. Blood flow (BF) and blood volume (BV) were calculated by DC.

The images of each patient were transferred to an Advanced Vitrea workstation (MS algorithm, Toshiba, Tokyo, Japan) and a GE ADW4.5 workstation (DC algorithm, GE, Boston, MA, USA). The images were retrospectively analyzed by two senior abdominal radiologists with more than 10 years of experience. Neither radiologist was aware of the clinical data or the type of algorithm used. Both radiologists were blinded to the clinical data and the type of algorithm used. According to the software recommendation, the threshold was set to 0–120 HU. The central part of the aorta at the pancreatic level was selected as the input artery and the spleen was selected as the reference organ. The regions of interest (ROIs) were placed on sections of the maximum diameter of the head, body, and tail of the pancreas; the measured area was about 1–2 cm^2^, while avoiding blood vessels as far as possible. The perfusion parameters BF and BV were recorded. BV cannot be directly calculated using the MS method, so an alternative equivalent BV is calculated using the Patlak plot method proposed by Goh et al.^6^ For each of the above‐mentioned parameters, the target tissues were measured three times in the axial, sagittal, and coronal planes, and the average value was taken.

### 2.4 Statistical analysis

The CTP parameters were expressed as the ($\bar{x}$± s). The statistical analysis was performed using SPSS v19.0 and Medcalc v11.4 statistical software programs. Single‐factor analysis of variance was used to compare the differences in perfusion parameters between the pancreatic head, body, and tail. The paired *t*‐test was performed to compare and analyze the differences in CTP parameters between the two algorithms. Pearson linear correlation analysis was performed to assess the correlation of the CTP parameters between the two algorithms. The Bland–Altman analysis was used to analyze the consistency of the CTP parameters between the two algorithms. Intraclass correlation coefficients were performed to compare the consistency between the two observers. Interobserver consistency (ICC) was considered good if it was greater than 0.6. *p*‐Values of less than 0.05 were considered to indicate statistically significant differences.

## 3 RESULTS

### 3.1 General information

A total of 68 patients underwent CTP imaging of the pancreas in our hospital during the study period. After the application of the selection criteria, a total of 57 consecutive patients (37 men and 20 women) with a mean age of 51.2 ± 10.2 years were included in the present study. Of these 57 subjects, 30 subjects had a normal pancreas and 27 subjects had acute interstitial pancreatitis confirmed by a laboratory, imaging, and clinical examinations.^20^

### 3.2 Comparison of CTP parameters between the MS and DC algorithms

**FIGURE 2:**
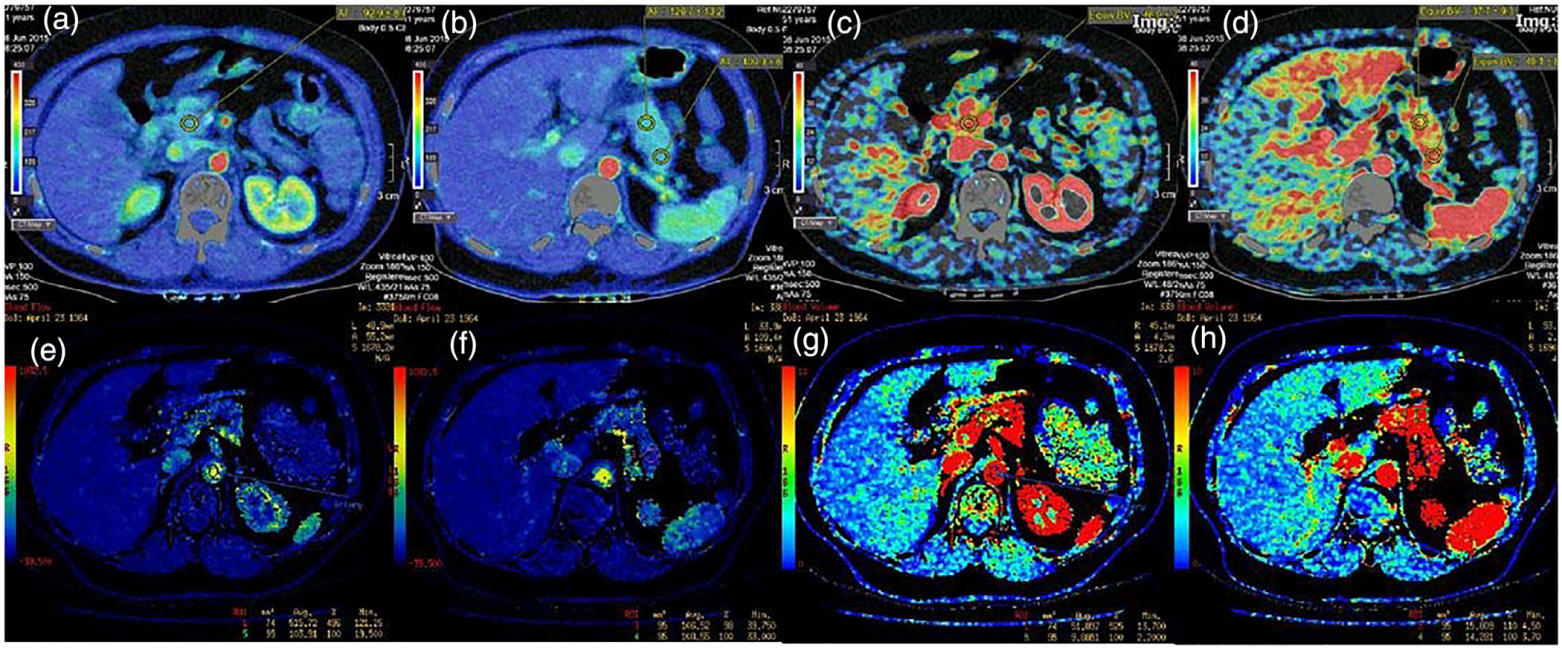
A 61‐year‐old man with a normal pancreas. (a and b): Blood flow in the pancreatic head, body, and tail regions was measured using the maximum slope (MS) method. (c and d): Blood volume in the pancreatic head, body, and tail regions was detected by the MS method. (e and f): Blood flow in the pancreatic head, body, and tail regions was treated using the deconvolution (DC) method. (g and h): Blood volume in the pancreatic head, body, and tail regions was measured by the DC method

**FIGURE 3:**
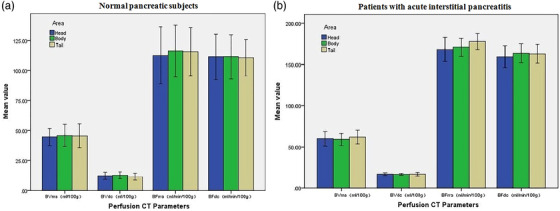
Blood flow (BF) and blood volume (BV) in the pancreatic head, body, and tail regions in (a) normal pancreatic subjects and (b) patients with acute interstitial pancreatitis.

As shown in Figure 2, BV and BF of each subject at the head, body, and tail of the pancreas were measured using the MS and DC algorithms. The mean BV and BF values for the three pancreatic regions obtained using the two algorithms were presented in Table 1 and Figure 3. Among the 30 normal pancreatic subjects, the BV values in the three pancreatic regions were markedly higher in the case of the MS algorithm than that in the case of the DC algorithm (*t* = 39.35, *p* < 0.001). In the same subjects, the BF values in the three pancreatic regions were slightly higher for the MS algorithm than that for the DC algorithm (*t* = 2.19, *p* = 0.031). The BV and BF values had no significant differences between the different regions of the normal pancreas for either algorithm (BV: *F* = 0.19, *p* = 0.827 [MS] and *F* = 0.23, *p* = 0.788 [DC]; BF: *F* = 0.069, *p* = 0.348 [MS], and *F* = 0.025, *p* = 0.975 [DC]).

**TABLE 1:** Comparison of the maximum slope (MS) and deconvolution (DC) algorithms for the calculation of perfusion parameters in different target tissues ($\bar{x}$± s)

		**BV (ml·100 g^–1^)**		**BF (ml/min^–1^·100 g^–1^)**	
		**MS**	**DC**	**MS**	**DC**
Normal pancreas (*n* = 30)	Head	45.55 ± 7	12.29 ± 3	112.61 ± 24	111.32 ± 19
	Body	45.90 ± 9	12.70 ± 3	116.26 ± 22	111.39 ± 18
	Tail	45.61 ± 10	11.63 ± 3	115.58 ± 20	110.47 ± 15
		*t* = 39.35, *p* < 0.001		*t* = 2.19, *p* = 0.031	
Acute pancreatitis (*n* = 27)	Head	60.02 ± 9	16.61 ± 2	168.29 ± 15	59.31 ± 13
	Body	59.16 ± 7	16.33 ± 1	170.82 ± 11	163.55 ± 12
	Tail	61.89 ± 9	16.53 ± 2	172.94 ± 10	162.86 ± 11
		*t* = 54.14, *p* < 0.001		t = 8.45, *p* < 0.001	

Abbreviations: BF, blood flow; BV, blood volume.

Similarly, the BV values obtained using the MS methods were markedly higher than those obtained using the DC methods among the 27 patients with acute pancreatitis (*t* = 54.14, *p* < 0.001). Furthermore, the BF values were higher with the MS methods than that with the DC methods (*t* = 8.45, *p* < 0.001). However, the BV and BF values had no significant differences between the different pancreatic regions for either method (BV: *F* = 0.765, *p* = 0.469 [MS] and *F* = 2.502, *p* = 0.565 [DC]; BF: *F* = 1.188, *p* = 0.629 [MS], and *F* = 0.956, *p* = 0.389 [DC]). Besides, the consistency between the two radiologists was good (ICC ≥ 0.9).

### 3.3 Pearson linear correlation analysis

Pearson linear correlation analysis showed that the BF and BV values of head, body, and tail of the pancreas were well correlated between the MS algorithm and DC algorithm in 30 subjects with the normal pancreas (*p *< 0.05, *r* > 0.6), and the correlation of the BF values was better than that of the BV values. Furthermore, in 27 patients with acute pancreatitis, the BF and BV values showed a good correlation between the two algorithms (*p *< 0.05, *r* > 0.7).

### 3.4 Bland–Altman analysis

**FIGURE 4:**
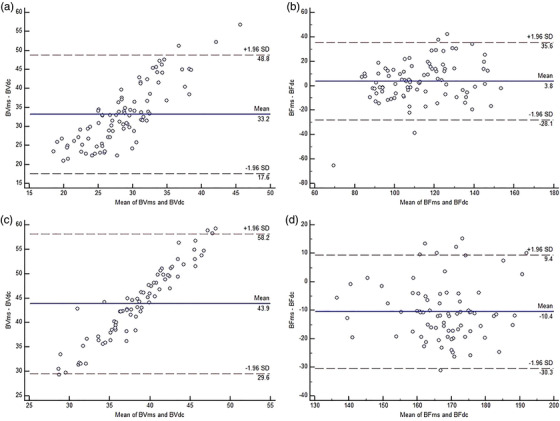
Bland–Altman analysis charts of the perfusion parameters (blood flow [BF] and blood volume [BV]) in the normal pancreas (a and b) and acute pancreatitis groups (c and d) were obtained using the deconvolution (DC) and maximum slope (MS) methods. The intermediate solid line represents the mean difference of the actual measurement values calculated using the two methods. The upper and lower symmetrical horizontal dotted lines represent the upper and lower 95% conformance boundaries of the mean difference, respectively

In the 30 subjects with the normal pancreas (90 ROIs), the mean difference of BV value obtained using the two algorithms was 33.2 ml/100 g. Most (87/90) of the BV data were distributed within the interval of 95% consistency (17.6–48.8). There were 3 (3.3%) outside data points. The absolute maximum difference between the two methods was 51.69 (Figure 4a). In the same subjects, the mean difference of the BF value was 3.8 ml/min^–1^·100 g^–1^ for the two algorithms. Most (86/90) of the BF data were distributed in the interval of 95% consistency (−28.1 to 35.1); there were 4 (4.4%) outside data points. The absolute maximum difference between the two methods was 41.44 (Figure 4b).

Among the patients with acute pancreatitis (81 ROIs), the mean difference of BV value was 43.9 ml/100 g for the two algorithms. Most (76/81) of the data were distributed in the interval of 95% consistency (29.6–58.2) and the number of outside data points was 5 (6.2%). The absolute maximum difference between the two methods was 60.93 (Figure 4c). The mean difference in BF value was 10.4 mL/min^–1^·100 g^–1^ for the two algorithms. Most (74/81) of the data were distributed in the interval of 95% consistency (−30.3 to 9.4) and the number of outside data points was 7 (8.6%). The absolute maximum difference between the two methods was 34.18 (Figure 4d).

## 4 DISCUSSION

### 4.1 Correlation and consistency between the MS and DC methods

In this study, we found that the BV and BF values had no significant differences between different pancreatic regions for either algorithm (Table 1). This was consistent with the previous reports. For instance, Zhu et al.^21^ compared the BF values of the pancreatic head, body, and tail in 32 subjects with normal pancreas and prove that there are no significant differences in BF values between the different pancreatic regions. Besides, Delrue et al.^22^ evaluated the normal pancreatic head, body, and tail in 20 subjects and confirmed that BV and BF had no significant differences between the different pancreatic regions.

Furthermore, the results of Pearson linear correlation analysis indicated that there was a positive correlation between BV and BF values of pancreatic head, body, and tail of the two algorithms. Kishimoto^23^ compared the BV and BF values of nine canine pancreases between the MS and DC methods and confirmed a good correlation between the two methods. This was consistent with the results of the present study, but they do not analyze the consistency of the results between the two methods. Kanda et al.^9^ compared both the correlation and consistency of the BF of the hepatic artery and portal vein between the MS and DC methods in 88 patients and the results show a good correlation between the two methods, but the values obtained using the MS method are significantly higher than those obtained using the DC methods. Similarly, our study showed that the BV and BF values calculated using the MS method were higher than those calculated using the DC methods. In particular, the BV values obtained using the MS method were three times those obtained using the DC method. The reason might be that the MS algorithm assumed that there was no outflow of contrast agent for a short period before the maximum TDC slope of the tissue was reached after the injection of the contrast agent. However, in the actual physiological state, contrast agent outflow is present. Thus, the perfusion parameters obtained using the MS method are higher than those in the actual physiological state. In contrast, the DC algorithm does not assume the tissue perfusion model and the result is closer to the actual physiological state.^9^, ^24^

Bland–Altman statistical analysis was used to evaluate the consistency of the perfusion parameters between the MS and DC methods and the results showed that the mean difference value of BV and BF values in both the subjects with the normal pancreas and those with pancreatitis were not equal to 0, indicating that the values of the perfusion parameters measured by the two mathematical algorithms were not consistent. Besides, within the bounds of the 95% consistency, the difference in the perfusion parameters between the two algorithms (the absolute value of the maximum difference) was very large, which was not acceptable in clinical practice. This highlighted the impact of the mathematical algorithms on the calculation of perfusion parameters. Due to the inconsistency of the perfusion parameters obtained by different algorithms, it should be noted that the thresholds for perfusion parameters based on the DC method could not be applied to the MS method when performing clinical disease analysis and multi‐center data exchange research, and vice versa. The results of this study remind us that we should pay attention to the algorithm used when using perfusion parameters to determine the severity of pancreatitis and monitor the efficacy. Because the perfusion parameters obtained by different algorithms are inconsistent, the threshold values of the perfusion parameters obtained by the DC method should not be used to judge the severity of pancreatitis measured by the MS method and monitor the efficacy. The conclusion of this study application had certain significance for clinical practice, the application of perfusion parameters in clinical practice, especially the threshold to differentiate benign tumors and monitoring curative effect should first clear the perfusion parameters adopted by the mathematical algorithm, might not be based on the DC method of perfusion parameters threshold directly to the MS method was used to measure of benign and malignant tumor, curative effect monitoring clinical situations.

### 4.2 Limitations of the study

First, the locations and sizes of the ROIs might affect the measurement results. Second, in this study, the scanning initiation time was 10 s after contrast injection, and no individual factors such as cardiac function were taken into consideration. However, the MS method was more affected by cardiac parameters (such as cardiac output) compared with the DC method.^24^ Third, the high flow rate of the contrast agent might affect the results. Therefore, they were not transferable to routine clinical practice.

## 5 CONCLUSIONS

In conclusion, the BF and BV values measured by the MS and DC algorithms had a good correlation but were not consistent. In clinical practice, the algorithm used to acquire these data should be noted, as the values obtained using the two algorithms were not interchangeable.

## CONFLICT OF INTEREST

The authors declare that there is no conflict of interest that could be perceived as prejudicing the impartiality of the research reported.

## AUTHOR CONTRIBUTIONS

Xiufen Jia designed the study. Kehua Pan prepared figures and wrote the manuscript. Hongqing Wang, Xiaoyu Chen and Xiaocui Ye helped to collect the clinical data and also statistical analysis. Zhao Zhang and Xiao Chen helped to revise minor errors and proofreading. All authors reviewed and approved the final manuscript.
